# Non-HDL cholesterol is a good predictor of the risk of increased arterial stiffness in postmenopausal women in an urban Brazilian population

**DOI:** 10.6061/clinics/2017(02)07

**Published:** 2017-02

**Authors:** Rafael de Oliveira Alvim, Carlos Alberto Mourao, Géssica Lopes Magalhães, Camila Maciel de Oliveira, José Eduardo Krieger, José Geraldo Mill, Alexandre Costa Pereira

**Affiliations:** I Universidade Federal do Espírito Santo, Departamento de Saúde Pública, Vitória/ES, Brazil; II Faculdade de Medicina da Universidade de São Paulo, Instituto do Coração (INCOR), Laboratório de Genética e Cardiologia Molecular, São Paulo/SP, Brazil; III Universidade Federal de Juiz de Fora, Departamento Fisiologia, Juiz de Fora/MG, Brazil

**Keywords:** Non-HDL-C, Arterial Stiffness, Dyslipidemia, Menopause

## Abstract

**OBJECTIVES:**

Increased arterial stiffness is an important determinant of the risk of cardiovascular disease. Lipid profile impairment, especially hypercholesterolemia, is associated with stiffer blood vessels. Thus, the aim of this study was to determine which of the five circulating lipid components (high-density lipoprotein cholesterol (HDL-C), non-high-density lipoprotein cholesterol (non-HDL-C), low-density lipoprotein cholesterol (LDL), total cholesterol (TC) and triglycerides) is the best predictor of increased arterial stiffness in an urban Brazilian population.

**METHODS:**

A random sample of 1,662 individuals from the general population of Vitoria, Brazil (25-64 years), was selected, and lipid components were measured using standard methods. Pulse wave velocity was measured using a non-invasive automatic device, and increased arterial stiffness was defined as a pulse wave velocity ≥10 m/s.

**RESULTS:**

In men, only total cholesterol (OR=1.59; CI=1.02 to 2.48, *p*=0.04) was associated with the risk of increased arterial stiffness. In women, HDL-C (OR=1.99; CI=1.18 to 3.35, *p*=0.01) and non-HDL-C (OR=1.61; CI=1.01 to 2.56, *p*=0.04) were good predictors of the risk of increased arterial stiffness. However, these associations were only found in postmenopausal women (OR=2.06; CI=1.00 to 4.26, *p*=0.05 for HDL-C and OR=1.83; CI=1.01 to 3.33, *p*=0.04 for non-HDL-C).

**CONCLUSION:**

Our findings indicate that both HDL-C and non-HDL-C are good predictors of the risk of increased arterial stiffness in postmenopausal women in an urban Brazilian population and may be useful tools for assessing the risk of arterial stiffness.

## INTRODUCTION

Increased arterial stiffness is an important determinant of cardiovascular disease (CVD) risk 1. Several epidemiological studies have reported that increased arterial stiffness predicts morbidity and mortality independently of other cardiovascular risk factors 2,3. Stiffness of the large arteries, which is measured using carotid-femoral pulse wave velocity (PWV) - the gold standard method for assessing this parameter - has been associated with measures of subclinical atherosclerosis and CVD 4. Furthermore, clinical studies have shown that arterial stiffness increases with age or in several pathological processes, such as hypertension, metabolic syndrome, chronic renal disease, hypercholesterolemia, and menopause 5-10.

Changes in the lipid profile, especially hypercholesterolemia, may contribute to changes in vascular stiffness 9. Several studies have recently investigated the association between arterial stiffness and lipid profile-related parameters, such as non-high-density lipoprotein cholesterol (non-HDL-C), low density lipoprotein cholesterol (LDL-C) and the non-HDL/HDL-C ratio 11-13. However, the results reported to date are far from conclusive because the results of only 10% of the studies on this subject suggested that a positive association exists between these variables, while most of the studies failed to find such a correlation between the variables 14. In addition, the results of the studies showing that any serum lipid parameter is a superior predictor of the risk of increased arterial stiffness compared to other lipid profile parameters are still inconclusive.

Based on this scenario and on the clinical applicability of lipid profile and arterial stiffness measurements, the aim of this study was to determine which of the five measurements recommended to assess cardiovascular risk (HDL-C, non-HDL-C, LDL-C, total cholesterol (TC) and triglycerides) is the best predictor of the risk of increased arterial stiffness in an urban Brazilian population. Moreover, we also assessed the possible influence of menopause on PWV values.

## METHODS

### Subjects

A study of the risk factors for CVDs was performed in the urban population of Vitoria, Brazil, using the WHO-MONICA Project guidelines 15. The study design was based on a cross-sectional research methodology and was developed by surveying and analyzing socioeconomic and health data in a probabilistic sample of residents from the municipality of Vitoria, Espírito Santo, Brazil. The objective of the sampling plan was to ensure that the research was socioeconomically, geographically, and demographically representative of the residents of this municipality. Residents of the city of Vitoria ranging from 25-64 years of age were included in the study. According to the census conducted by the IBGE Foundation in 1996, the resident population of Vitoria included 265,874 inhabitants. Sampling was performed in four stages as follows: by district, by IBGE census sector, by drawing lots to choose homes, and by birthday to choose an individual from each home. The survey was conducted with only one resident (chosen according to the nearest birthday after homes were randomly selected) from the selected home who was within the aforementioned age range of the study. The draw was conducted using a randomization mechanism. A total of 2,268 residential homes were selected and visited. The research purposes were explained to the individual selected in his/her home, and he/she was invited to participate in the study after providing his/her written consent. The selected individuals were asked to visit the Cardiovascular Investigation Clinic of the University Hospital within 2 weeks after selection so that their tests could be performed. Among the total sample, 1,662 individuals (761 men and 901women) visited the clinic. This study was approved by the Ethics Committee for Research on Human Subjects of the Espírito Santo Federal University and the National Ethics Committee for Human Research (CONEP Register Number 4599).

### Anthropometrical Investigations

Anthropometric parameters were measured according to a standard protocol 16. Body weight was measured to the nearest 0.1 kg on a calibrated scale, and height was measured to the nearest 0.5 cm using a wall-mounted stadiometer. Waist circumference was measured at the mean point between the lowest rib margin and the iliac crest at the maximum point of normal expiration while the subject stood 17. Body mass index (BMI) was calculated as body weight (kg) divided by height squared (m^2^).

### Biochemical Measurements

Blood glucose levels, triglyceride levels and lipoprotein fractions were assayed with standard techniques using 12-h fasting blood samples 18. Non-HDL-C was calculated by subtracting HDL-C from TC. The following biochemical parameter results were categorized as abnormal: a TC level ≥240 mg/dL, a triglyceride level ≥200 mg/dL, an LDL-C level ≥160 mg/dL, an HDL-C level ≤39 mg/dL, and a non-HDL-C level ≥190 mg/dL 19. Diabetes mellitus was diagnosed based on the presence of a fasting glucose level ≥126 mg/dL and/or antidiabetic drug use 20, and hyperlipidemia was defined as a TC level ≥240 mg/dL, an LDL-C level ≥160 mg/dL, and/or hypolipidemic drug use 21.

### PWV Determination

Carotid-femoral PWV was measured with the patient in the supine position with an automatic and validated device (Complior; Artech Medical, France) operated by an experienced observer who was blinded to the patients’ clinical characteristics. Briefly, common carotid artery and femoral artery pressure waveforms were recorded non-invasively using pulse-sensitive transducers. PWV was measured within a period ranging from 10–15 seconds, and carotid-femoral distance was used to assess PWV. The distance between the recording sites (D) was measured, and PWV was calculated automatically as PWV=D/t, where (t) is the pulse transit time. The measurements were repeated over 10 different cardiac cycles. According to recent recommendations, increased arterial stiffness was defined as a PWV≥10 m/s 22. The validation of this automatic method and its reproducibility have been described previously 23.

### Assessment of Menopausal Status

Information regarding the menopausal statuses of the women in the present study was evaluated using a questionnaire that was completed by each participant in an interview during the clinic visit. The questionnaire was based on the WHO-MONICA epidemiological instrument 15 and was administered and filled out by research assistants who were specially trained for the task.

### Statistical Analysis

Categorical variables are presented as percentages, whereas continuous variables are presented as the mean±standard deviation. Binary logistic regression analysis was performed to evaluate the associations between lipid profile parameters (HDL-C, non-HDL-C, LDL-C, TC and triglycerides) and arterial stiffness, allowing for covariates (mean blood pressure and age). The data are presented as odds ratios (ORs) and 95% confidence intervals (CIs). The same analysis was also performed second time only for women and was stratified by menopausal status (premenopausal and postmenopausal women). Statistical analyses were conducted using IBM SPSS Statistics for Windows, Version 19.0. Armonk, NY, USA, with the level of significance set at 5%.

## RESULTS

The results are presented such that they emphasize the relationship between lipid components and increased arterial stiffness in women because some lipid components were strongly associated with increased arterial stiffness in this group, especially in postmenopausal women.

Stratified by sex, the demographic data related to age, BMI, waist circumference, fasting glucose, PWV, TC, HDL-C, LDL-C, non-HDL-C, very-low-density lipoprotein cholesterol (VLDL), triglycerides and the presences of diabetes, smoking, hypertension, hyperlipidemia and increased arterial stiffness for the entire sample are summarized in Table 1. The demographic data stratified by the menopausal statuses of the women are presented in Table 2.

### Association Between Lipid Profile Parameters and Increased Arterial Stiffness

In men, only TC (OR=1.59; CI=1.02 to 2.48, *p*=0.04) was significantly associated with increased arterial stiffness (Table 3). However, in women, both HDL-C (OR=1.99; CI=1.18 to 3.35, *p*=0.01) and non-HDL-C (OR=1.61; CI=1.01 to 2.56, *p*=0.04) were known to be good predictors of the risk of increased arterial stiffness (Table 3). When stratified by menopausal status, the same analysis showed that this association was significant only for postmenopausal women (OR=2.06; CI=1.00 to 4.26, *p*=0.05 for HDL-C and OR=1.83; CI=1.01 to 3.33, *p*=0.04 for non-HDL-C) (Table 4). Accordingly, none of the lipid profile parameters were significantly associated with increased arterial stiffness in premenopausal women.

## DISCUSSION

The main finding of our study was that there were positive associations between HDL-C and non-HDL-C and increased arterial stiffness in postmenopausal women. These results were not replicated in men and premenopausal women. In addition, postmenopausal women displayed higher LDL-C, triglyceride, TC, non-HDL-C and PWV levels than premenopausal women.

In the last few decades, researchers have noted an association between aging and dyslipidemia. In men, lipid profiles become more unfavorable because of the decreases in testosterone levels that occur with age. Similarly, in women, menopause is associated with a more atherogenic lipid profile than premenopausal status 24. Studies have shown associations between the onset of menopause and the increased prevalence of cardiovascular risk factors. The lack of estrogen facilitates the development of diabetes, hypertension, obesity and dyslipidemia 25. In our study, we also showed that a decrease in estrogen predisposes individuals to developing increased arterial stiffness, which is an additional and independent risk factor for CVD. Experimental studies have demonstrated that estrogen is highly effective in preventing LDL-C and VLDL-C oxidation 26. In addition, endogenous estrogen production in premenopausal women is associated with low LDL-C levels and high HDL-C levels 27. Mogarekar and Kulkarni 28 showed that postmenopausal women had significantly increased serum triglycerides and small dense LDL-C levels, as well as significantly decreased HDL-C and paraoxonase-1 levels, compared to premenopausal women. These findings may explain the low prevalence of CVD in premenopausal women. In the present study, postmenopausal women displayed worse lipid profiles and higher prevalences of hypertension, diabetes and increased arterial stiffness than premenopausal women. Thus, it is possible that the development of cardiometabolic disorders can be partially explained by deficient or absent estrogen production.

Increased central arterial stiffness is an important determinant of CVD risk 1, and previous studies have demonstrated that an association exists between hypercholesterolemia and stiffer blood vessels 9. In our study, the lipid profile was associated with increased arterial stiffness in postmenopausal women; i.e., high HDL-C and non-HDL-C levels were associated with increased arterial stiffness. As previously described, estrogen plays a key role in lipid metabolism and cardiovascular risk among women 25,27. Thus, our data suggest that increased arterial stiffness in postmenopausal women may result from the lipid profile parameter worsening associated with decreased estrogen levels.

Several studies have focused on the relationship between the lipid profile and arterial stiffness. Holewijn et al. 11 studied 1517 individuals aged 50-70 years and showed that non-HDL-C was superior to LDL-C with respect to identifying individuals with compromised cardiovascular phenotypes, including individuals with high arterial stiffness, from the general population, results that partially corroborated ours. In contrast, Zhao et al. 13 studied a middle-aged and elderly Chinese population and showed that the non-HDL-c/HDL-C ratio was superior to traditional lipid variables with respect to estimating arterial stiffness risk. Finally, Wang et al. 12 studied 2375 individuals aged 40-96 years and showed that only LDL-C and HDL-C were independently associated with aortic stiffness. However, that study did not evaluate the role of non-HDL-C with respect to the risk of aortic stiffness. As stated above, we were limited with respect to our ability to compare our results with those of previous studies in the literature due to the variety of methods used to assess arterial stiffness in those studies and the differences between those studies and ours with regard to the statistical methods used for analyses.

In the present study, non-HDL-C was a good predictor of the risk of increased arterial stiffness in postmenopausal women belonging to an urban Brazilian population. Our data were partially corroborated by the results of several population studies demonstrating that non-HDL-C is a better marker of CVD risk than LDL-C alone 29,30. Another important finding of our study is that the association between the lipid profile and increased arterial stiffness was significant only in postmenopausal women, a finding suggestive of the impact of estrogen on lipid metabolism 26,27.

Our study had some limitations. First, we did not obtain measurements of follicle stimulating hormone (FSH) levels; therefore, we were unable to make a laboratory diagnosis of menopause. Second, this study was a cross-sectional analysis. Therefore, a causal relationship between increased arterial stiffness and poor lipid profiles could not be established. Third, in our study, some women were treated with postmenopausal hormone replacement therapy. Fourth, hypolipidemic therapy can decrease arterial stiffness 31, and some participants enrolled in the our study use lipid-lowering drugs, which may have slightly influenced our findings.

In summary, this study showed that non-HDL-C may be a good predictor of the risk of increased arterial stiffness in postmenopausal women. Thus, we surmise that non-HDL-C may a useful tool for the assessing the risk of arterial stiffness during the postmenopausal period.

## AUTHOR CONTRIBUTIONS

Alvim RO, Magalhães GL and Mourao-Junior CA participated in the design of the study, performed the statistical analysis and drafted the manuscript. De Oliveira CM and Mill JG contributed to the acquisition and interpretation of the data. Krieger JE contributed to the conception and design of the study. Pereira AC conceived the study, participated in its design, and coordinated and assisted with the drafting of the manuscript. All authors read and approved the manuscript.

## Figures and Tables

**Table 1:** Characteristics of the subjects in the sample.

Characteristics	Men	Women
**N**	761	901
**Age, years**	44.7±10.9	44.8±10.7
**BMI, kg/m^2^**	25.9±4.0	26.6±5.5
**Waist circumference, cm**	89.1±10.9	83.6±12.9
**Fasting glucose, mg/dL**	105.3±28.2	104.0±33.9
**Smoking (%)**	25.6	19.5
**Hypertension (%)**	47.6	38.1
**Diabetes (%)**	7.1	8.2
**Hyperlipidemia (%)**	26.9	25.9
**Increased arterial stiffness (%)**	19.3	12.1
**PWV, m/s**	7.9±2.3	7.4±2.1
**TC, mg/dL**	213.3±50.8	215.2±44.9
**HDL-C, mg/dL**	42.3±12.3	48.0±11.9
**LDL-C, mg/dL**	140.4±39.4	143.6±39.8
**Non-HDL-C, mg/dL**	171.6±55.2	167.5±47.6
**VLDL-C, mg/dL**	29.2±24.7	22.8±15.1
**Triglycerides, mg/dL**	163.5±175.9	118.4±85.8

BMI, body mass index; PWV, pulse-wave velocity; TC, total cholesterol; HDL-C, high-density lipoprotein cholesterol; LDL-C, low-density lipoprotein cholesterol; Non-HDL-C, non-high-density lipoprotein cholesterol; VLDL-C, very-low-density lipoprotein cholesterol.

Hypertension: mean systolic blood pressure ≥140 mmHg, mean diastolic blood pressure ≥90 mmHg and/or anti-hypertensive drug use.

Diabetes: fasting glucose ≥126 mg/dL and/or hypoglycemic drug use.

Increased arterial stiffness: pulse-wave velocity (PWV) ≥10 m/s.

Hyperlipidemia: TC≥240 mg/dL, LDL-C≥160 mg/dL, and/or hypolipidemic drug use.

Continuous data are expressed as the mean±standard deviation.

**Table 2:** Clinical characteristics of the women stratified by menopause status.

Characteristics	Premenopausal Women	Postmenopausal Women	*p-*value
**Age, y**	40.2±8.9	54.8±6.7	**<0.001**
**BMI, kg/m^2^**	26.2±5.6	27.4±5.1	**0.002**
**Waist circumference, cm**	81.7±12.5	87.3±12.6	**<0.001**
**Fasting glucose, mg/dL**	99.4±22.5	114.1±49.6	**<0.001**
**PWV, m/s**	7.0±1.9	8.3±2.2	**<0.001**
**CT, mg/dL**	203.8±40.5	239.9±45.2	**<0.001**
**LDL-C, mg/dL**	135.2±36.6	161.6±41.2	**<0.001**
**HDL-C, mg/dL**	47.9±12.5	48.6±10.9	0.39
**Non-HDL-C, mg/dL**	156.5±42.5	191.2±50.3	**<0.001**
**Triglycerides, mg/dL**	106.6±78.1	145.0±96.9	**<0.001**
**Smoking (%)**	19.8	18.6	0.42
**Increased arterial stiffness (%)**	6.6	24.4	**<0.001**
**Diabetes (%)**	5.1	14.7	**<0.001**
**Hypertension (%)**	28.1	60.7	**<0.001**
**Hyperlipidemia (%)**	18.4	42.7	**<0.001**

Continuous data are expressed as the mean±standard deviation.

BMI, body mass index; PWV, pulse-wave velocity; TC, total cholesterol; HDL-C, high-density lipoprotein cholesterol; LDL-C, low-density lipoprotein cholesterol; Non-HDL-C, non-high-density lipoprotein cholesterol.

Hypertension: mean systolic blood pressure ≥140 mmHg, mean diastolic blood pressure ≥90 mmHg and/or anti-hypertensive drug use.

Diabetes: fasting glucose ≥126 mg/dL and/or hypoglycemic drug use.

Increased arterial stiffness: pulse wave velocity (PWV)≥10 m/s.

Hyperlipidemia: TC≥240 mg/dL, LDL-C≥160 mg/dL, and/or hypolipidemic drug use.

**Table 3:** Analysis of increased arterial stiffness according to the lipid profiles of the sample.

Variables	OR (95% CI), *p-*value	
	**Men**	**Women**
**TC** (≥240 *versus* ≤239 mg/dL)	1.59 (1.02 to 2.48), **0.04**	1.37 (0.86 to 2.18), 0.19
**Triglycerides** (≥200 *versus* ≤199 mg/dL)	1.54 (0.97 to 2.46), 0.07	1.67 (0.95 to 2.92), 0.07
**HDL-C** (≤39 *versus* ≥40 mg/dL)	0.98 (0.64 to 1.53), **0.94**	1.99 (1.18 to 3.35), **0.01**
**LDL-C** (≥160 *versus* ≤159 mg/dL)	1.10 (0.69 to 1.74), 0.70	1.15 (0.71 to 1.87), 0.56
**Non-HDL-C** (≥190 *versus* ≤189 mg/dL)	1.34 (0.87 to 2.06), 0.19	1.61 (1.01 to 2.56), **0.04**

TC, total cholesterol; HDL-C, high-density lipoprotein cholesterol; LDL-C, low-density lipoprotein cholesterol; Non-HDL-C, non-high-density lipoprotein cholesterol; PWV, pulse wave velocity.

Increased arterial stiffness = PWV≥10 m/s.

All analyses were adjusted for mean blood pressure and age.

*p*-values in boldface are significant.

OR, odds ratio; 95%CI, 95% confidence interval.

**Table 4:** Analysis of increased arterial stiffness according to the lipid profiles of the women stratified by menopause status.

Variables	OR (95% CI), *p-*value	
	Premenopausal Women	Postmenopausal Women
**TC** (≥ 240 *versus* ≤239 mg/dL)	1.28 (0.58 to 2.79), 0.54	1.37 (0.76 to 2.46), 0.30
**Triglycerides** (≥ 200 *versus* ≤199 mg/dL)	1.91 (0.76 to 4.80), 0.17	1.64 (0.81 to 3.31), 0.17
**HDL-C** (≤39 *versus* ≥40 mg/dL)	1.94 (0.89 to 4.23), 0.09	2.06 (1.00 to 4.26), **0.05**
**LDL-C** (≥160 *versus* ≤159 mg/dL)	1.23 (0.56 to 2.69), 0.61	1.01 (0.55 to 1.87), 0.98
**Non-HDL-C** (≥190 *versus* ≤189 mg/dL)	1.13 (0.52 to 2.47), 0.35	1.83 (1.01 to 3.33), **0.04**

TC, total cholesterol; HDL-C, high-density lipoprotein cholesterol; LDL-C, low-density lipoprotein cholesterol; Non-HDL-C, non-high-density lipoprotein cholesterol; PWV, pulse wave velocity.

Increased arterial stiffness = PWV≥10 m/s.

All analyses were adjusted for mean blood pressure and age.

*p*-values in boldface are significant.

OR, odds ratio; 95%CI, 95% confidence interval.
